# Motif 1 Binding Protein suppresses *wingless* to promote eye fate in *Drosophila*

**DOI:** 10.1038/s41598-020-73891-7

**Published:** 2020-10-14

**Authors:** Akanksha Raj, Anuradha Venkatakrishnan Chimata, Amit Singh

**Affiliations:** 1 Department of Biology, University of Dayton, Dayton, OH 45469 USA; 2 Premedical Program, University of Dayton, Dayton, OH USA; 3 Center for Tissue Regeneration and Engineering (TREND), University of Dayton, Dayton, OH USA; 4 Integrative Science and Engineering (ISE), University of Dayton, Dayton, OH USA; 5 Center for Genomic Advocacy (TCGA), Indiana State University, Terre Haute, IN USA

**Keywords:** Developmental biology, Genetics, Neuroscience, Stem cells

## Abstract

The phenomenon of RNA polymerase II (Pol II) pausing at transcription start site (TSS) is one of the key rate-limiting steps in regulating genome-wide gene expression. In *Drosophila* embryo, Pol II pausing is known to regulate the developmental control genes expression, however, the functional implication of Pol II pausing during later developmental time windows remains largely unknown. A highly conserved zinc finger transcription factor, Motif 1 Binding Protein (M1BP), is known to orchestrate promoter-proximal pausing. We found a new role of M1BP in regulating *Drosophila* eye development. Downregulation of M1BP function suppresses eye fate resulting in a reduced eye or a “no-eye” phenotype. The eye suppression function of M1BP has no domain constraint in the developing eye. Downregulation of M1BP results in more than two-fold induction of *wingless* (*wg*) gene expression along with robust induction of Homothorax (Hth), a negative regulator of eye fate. The loss-of-eye phenotype of M1BP downregulation is dependent on Wg upregulation as downregulation of both M1BP and *wg*, by using *wg*^*RNAi*^, shows a significant rescue of a reduced eye or a “no-eye” phenotype, which is accompanied by normalizing of *wg* and *hth* expression levels in the eye imaginal disc. Ectopic induction of Wg is known to trigger developmental cell death. We found that upregulation of *wg* as a result of downregulation of M1BP also induces apoptotic cell death, which can be significantly restored by blocking caspase-mediated cell death. Our data strongly imply that transcriptional regulation of *wg* by Pol II pausing factor M1BP may be one of the important regulatory mechanism(s) during *Drosophila* eye development.

## Introduction

During organogenesis, the intricate process of gene regulation is facilitated by sequence specific factors and co-regulators, which turns on only a fraction of genes, while the rest of the genes are repressed or turned off. The dynamic temporal and spatial expression patterns of developmental control genes involves several checkpoints, starting from recruiting the general transcription machinery and RNA polymerase II (Pol II) to the gene promoter to initiate transcription. In higher eukaryotes, pausing of Pol II during early elongation phase of transcription serves as one of the regulatory mechanisms^1,2^. The genome-wide studies have shown that the regulation of Pol II activity near the transcription start site (TSS) is a widespread phenomenon in mammalian embryonic stem cells (ESCs) and *Drosophila*^3,4^. During *Drosophila* embryogenesis, most of the developmental control genes such as *Hox* genes, including the target gene promoters for various transcription factors and components of signaling pathways are transcriptionally paused. In *Drosophila* embryo, transcriptional regulation of three critical segmentation genes, *sloppy-paired-1* (*slp1*), *wingless* (*wg*) and *engrailed* (*en*) by Pol II pausing may play an important role in controlling the gene expression. However, its exact mechanism along later developmental time points is not completely understood^2,5–8^. We used *Drosophila* eye model to study the role of Pol II pausing during organogenesis such as development of adult organs/appendages from their imaginal primordium.

*Drosophila* eye is a highly versatile and tractable model system for understanding the gene regulatory mechanisms underlying complex developmental programs^9–13^. The adult *Drosophila* compound eye is a highly organized structure with approximately 600–800 ommatidia or unit eyes arranged in a hexagonal lattice^14^. Each ommatidium is comprised of approximately 20 cells including 8 photoreceptor (PR) cells, and non-neuronal cells like pigment cells, cone cells and bristles^9,14–16^. Of these 8 photoreceptor cells, there are outer photoreceptors R1-R6 and inner photoreceptors R7-R8. The adult eye develops from the larval eye-antennal imaginal disc^17^. The eye imaginal disc is specified during embryonic and early larval development by action of core retinal determination (RD) genes, *Pax6* homolog, *eyeless (ey)* and *twin of eyeless (toy)* and a network of mainly downstream transcription factors, *eyes absent (eya), sine oculis (so)*, *dachshund (dac), eyegone (eyg)* and *optix (opt)*^9,12,13,18–27^. Of these, *ey*, a *Drosophila* homolog of Pax-6, is involved in eye field specification whereas the downstream genes like *eya, so* and *dac* are involved in retinal determination and differentiation^15,23,27^. Loss-of-function of RD genes block the early eye development.

In the developing eye imaginal disc of *Drosophila* third instar larva, a synchronous wave of retinal differentiation is initiated from the posterior margin of the eye imaginal disc, which moves anteriorly, and is referred to as the Morphogenetic Furrow (MF)^14,16^. The MF, a transient indentation in the developing eye disc, sweeps progressively across the eye disc towards the anterior margin, resulting in the formation of uniformly spaced photoreceptor clusters behind the MF. This process of differentiation of retinal precursor cells to photoreceptor neurons is driven by combinatorial action of the evolutionarily conserved Hedgehog (Hh) and Decapentaplegic (Dpp) signaling pathways, which plays important role in initiation and progression of the MF^14,16,28–30^. Within each differentiating photoreceptor cluster, Hh and Dpp signaling activates the expression of proneural genes like *atonal (ato)* that encodes basic HLH proteins and specifies the R8 photoreceptor^31–33^. Followed by the R8 selection, sequential recruitment of R2/R5, R3/R4, R1/R6 and R7 occurs^9,22,34^. Another secreted protein Scabrous (Sca), which is expressed within and near the intermediate clusters in the MF, is required for the correct spacing of photoreceptor clusters^35,36^.

The differentiation of retinal neurons and MF progression is opposed by the secreted morphogen Wg (a homolog of mouse Wnt-1 gene), which is expressed on the antero-lateral regions of the eye imaginal disc^37–39^. Wg serves as a ligand for the highly conserved Wg/ WNT signaling pathway. In the developing eye, Wg, a morphogen, is involved in diverse functions like cell proliferation, cell death, and cell-fate specification^40–42^. Wg expression levels plays important role(s) in determining the eye versus head fate by antagonizing *dpp* and thereby suppressing retinal determination^11,13,15,38,42,43^. Wg regulates expression of another negative regulator of eye development, *homothorax (hth)*, a MEIS class gene with a highly conserved Meis-Hth (MH) domain and a homeodomain (HD), which is expressed uniformly anterior to the MF^44–48^ and suppresses the eye fate. Hence, ectopic upregulation of *wg* promotes head-specific fate by regulating MF progression during eye development^39,44,49^.

During eye development, one of the many functions of Wg signaling is to induce programmed cell death by activating the expression of *head involution defective (hid*), *reaper (rpr)*, and *grim* in ommatidia at the periphery of the eye during pupal stage^50,51^. In the developing larval eye field, apoptosis can be induced by a variety of stimuli like inappropriate levels of morphogens or extracellular signaling^52,53^. Ectopic induction of Wg signaling causes developmental or morphogenetic cell death in the larval eye imaginal disc^54^. This developmental cell death is caused by activation of caspases and is different from programmed cell death observed in the pupal retina^54,55^. The baculovirus anti-apoptotic protein P35 upon ectopic expression in the developing field can block caspase-dependent cell death^56^. P35 acts through inhibition of range of initiator to executioner class of caspases.

In *Drosophila*, the sequence-specific transcription factors, the GAGA factor (GAF) and the Motif 1 Binding Protein (M1BP) have been implicated in dictating Pol II pausing^57^. The regulatory mechanism associated with GAF exhibits greater transcriptional plasticity than M1BP. M1BP binds to a core promoter element called Motif 1 and has been shown to orchestrate promoter-proximal pausing in GAF-independent manner^57^. M1BP is highly conserved across the species and encodes a 55 kDa protein containing zinc-associated domain (ZAD) towards the N-terminus and five C_2_H_2_ zinc-fingers domains toward the C-terminus. *Drosophila* M1BP is functionally homologous to vertebrate zinc finger with a SCAN and a KRAB domain 3 (ZKSCAN3) transcription factors and shows structural similarity for the C-terminal C_2_H_2_ zinc-finger domains^58,59^. The M1BP binding site sequence was reported using bioinformatics and biochemical analysis^60^. In addition, these studies led to tracking of Motif 1 binding activity, their associated proteins, and a list of 2187 genes whose expression is affected by M1BP^57,61^.

Here, we demonstrate that *wg*, which encodes a ligand for the highly conserved Wg/WNT signaling pathway is one of the targets for M1BP mediated transcriptional regulation during *Drosophila* eye development. We show that downregulation of M1BP ectopically induces *wg* gene expression in the developing eye disc, which causes suppression of the eye fate and induction of developmental cell death. Our results clearly indicate that M1BP mediated transcriptional regulation of Wg signaling could be a key regulatory mechanism during *Drosophila* eye development. We found potential M1BP binding sites in regulatory regions of *wg* gene using bioinformatics. Furthermore, this relation was also observed in the wing imaginal discs.

## Materials and methods

### Fly stocks

Fly stocks used in this study are described in Flybase (https://flybase.bio.indiana.edu). We used *ey*-Gal4^62^, *eyg*-Gal4^26^, *bi*-Gal4 (BL 58,815)^63,64^, *dpp*-Gal4^65^, UAS-*M1BP*^*RNAi*^ (BL 41,937)^57^, UAS-*wg*^*RNAi*^ (BL 31,249)^66^, UAS-*hth*^*RNAi*^*, yw, hth*^*1422–4*^*/TM6B, Tb*^47^* dpp-*lacZ (BL 5528)^67^, *wg*-lacZ^62^, UAS-*P35*^56^. We used the wild-type Canton-S stock of *D. melanogaster* in this study. Fly stocks were maintained at 25 °C on the regular cornmeal, yeast, molasses food medium.

### Genetic crosses

We used Gal4/UAS TARGET system to misexpress the gene of interest^68^. All Gal4/UAS crosses were maintained at 18 °C, 25 °C and 29 °C, unless specified, to sample different induction levels^10^. The *ey*-Gal4 driver used in this study targets misexpression of inducible transgene *M1BP*^*RNAi*^ in the entire developing eye domain *(ey* > *M1BP*^*RNAi*^*)* of larval eye imaginal disc. To misexpress *M1BP*^*RNAi*^ in specific domains of the eye disc, different Gal4 drivers were used: *eyg*-Gal4 targets misexpression of transgene at the equator, *bi*-Gal4 selectively targets the expression of the transgene at the dorso-ventral (DV) eye margin^64^, *dpp*-Gal4 drives the expression of the transgene at the posterior margin of the eye disc^30^.

We also tested the gain-of-function of M1BP using the CRISPR/Cas9- based transcriptional activation approach^69^ to overexpress TRiP-CRISPR Overexpression (TRiP-OE) M1BP (BL 80231) in the *dpp* domain of the developing eye by crossing the *TOE M1BP* flies with *dpp-*Gal4*; dcas9-VPR* (BL 67045) flies, in which the tissue-specific Gal4 directs expression of a catalytically inactive dead Cas9 (dCas9) fused to a tripartite transcriptional activator domain, VP64-p65-Rta (VPR).

### Immunohistochemistry

Eye-antennal discs of wandering third instar larvae were dissected in 1 × phosphate buffered saline (PBS), fixed in 4% paraformaldehyde in PBS (fixative) for 20 min and washed in PBST (three times). The tissues were stained with a combination of antibodies following the standard protocol^39^. Primary antibodies used were rabbit anti*-*β-GAL (1:100; Cappel); rat anti-Elav (1:100), mouse anti-Wg (1:100; Developmental Studies Hybridoma Bank, DSHB), mouse anti- Dlg (1:100); mouse anti-Eya (1:100; DSHB), mouse anti-Dac (1:100; DSHB), goat anti-Hth (1:200; Santa Cruz), mouse anti-Sca (1:100), goat anti-Ato (1:50), rabbit anti-Dcp1 (1:150, Santa Cruz), mouse anti-pH3 (1:300, Cell Signaling). The discs were washed in PBST thrice for 10 min. Secondary antibodies used were donkey anti-rat IgG conjugated to Cy5 (1:250), donkey anti-rabbit IgG conjugated to Cy3 (1:300) or goat anti-mouse IgG conjugated to FITC (1:200) (Jackson Laboratories). The discs were mounted in Vectashield and photo-documented on a Fluoview 3000 Laser Scanning Confocal Microscope^70^. We took the images at 20 × magnification unless stated otherwise. We analyzed and prepared the final figures with images using Adobe Photoshop CS6 software.

### Adult eye imaging

Adult eye images were captured after freezing flies at − 20 °C for ~ 4 h. Images were taken on a MrC5 color camera mounted on an Axioimager.Z1 Zeiss Apotome using a Z-sectioning function of Axiovision software 4.6.3^71^. The final images were prepared using Adobe Photoshop CS6 software.

### Real time quantitative polymerase chain reaction (RT-qPCR)

Tissue was collected and homogenized in TRIzol Reagent (Invitrogen, Cat# 15596026). Total RNA was extracted following TRIzol protocol. Aqueous phase was transferred to RNA Clean & Concentrator-5 (Zymo research, Cat# R1013) columns and eluted in DNase/RNase-free water. Quality and quantity of isolated RNA was determined by Nanodrop 2000 spectrophotometer (Thermo Scientific). cDNA was synthesized from 1 µg of total RNA through Reverse Transcription reaction (RT) using first-strand cDNA synthesis kit (GE healthcare, Cat# 27926101). RT-qPCR was performed using BioRad iQ SYBR Green Supermix (Bio-Rad, Cat# 1708860) according to the standard protocol^72–74^ . Fold change was calculated using comparative CT method (2^−ΔΔCT^ method). The primers used are:

GAPDHFw5′-GGCGGATAAAGTAAATGTGTGC-3′.

GAPDHRev5′-AGCTCCTCGTAGACGAACAT-3′.

wgFw5′-TCAGGGACGCAAGCATAATAG-3′.

wgRev5′-CGAAGGCTCCAGATAGACAAG-3′.

### Statistics

Statistical analysis was performed using Microsoft excel software. The P-values were calculated using student’s t-test and the error bars represent Standard deviation from Mean. Statistical significance in each graph is shown by P-value: ***P < 0.001; **P < 0.01; *P < 0.05^75–78^.

## Results

### Downregulation of M1BP function suppresses the eye fate

**Figure 1:**
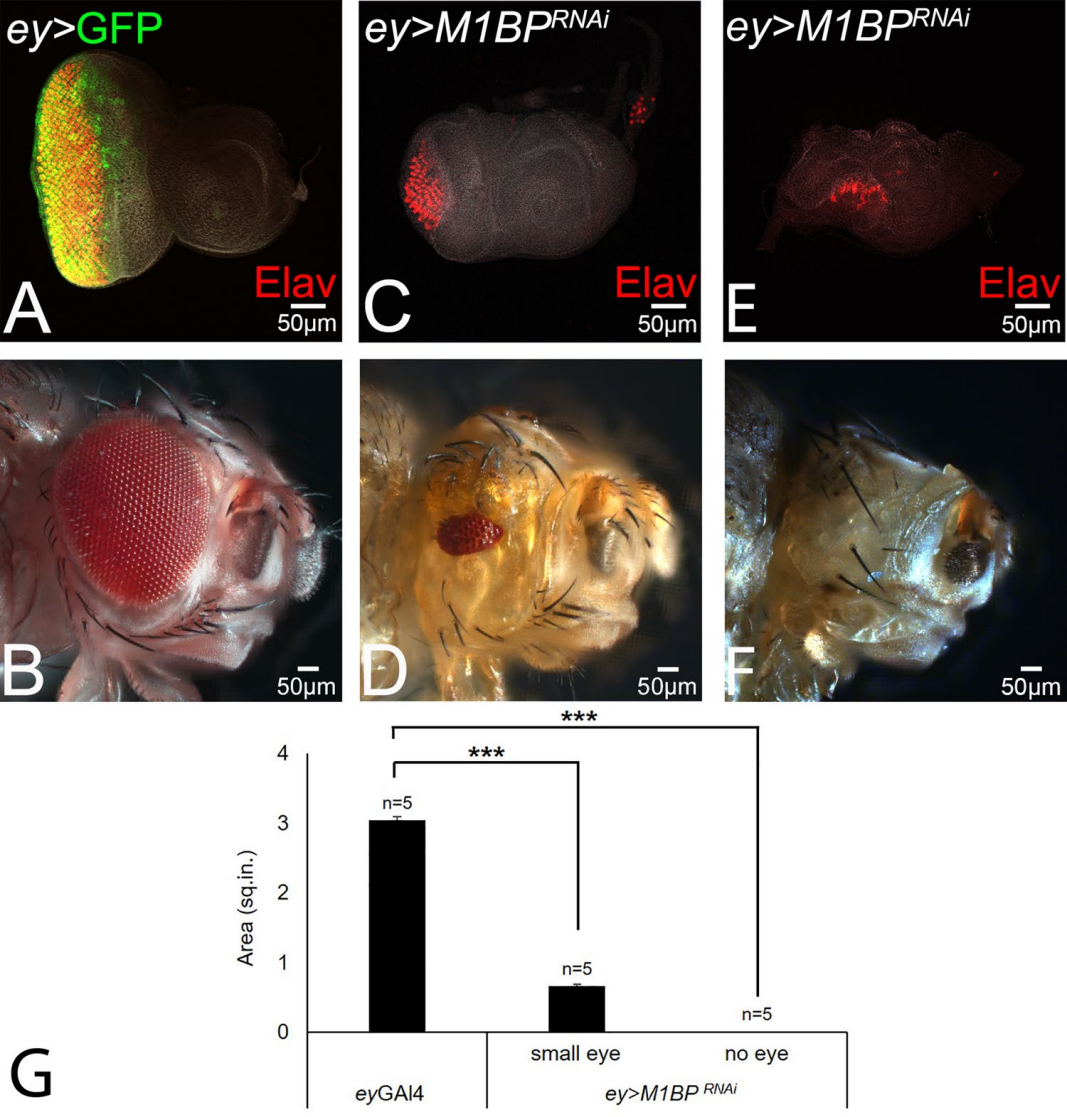
Downregulation of M1BP suppresses the eye fate during *Drosophila* eye development. (**A**) *ey*-Gal4 driven expression of UAS-GFP transgene in the eye. Note that *ey* > GFP (green) expressed in the entire eye field of third instar larval eye disc. Note that eye imaginal disc is stained with pan neuronal marker Elav (red) which marks the nuclei of retinal neurons and Dlg, a membrane specific marker, to mark outline of the tissue. (**B**) Adult eye. (**C**–**F**) Downregulation of M1BP in the eye by driving expression of UAS-M1BP^*RNAi*^* (ey* > *M1BP*^*RNAi*^*)* suppresses the eye fate as seen in (**C**,**E**) the eye imaginal disc and the (**D**,**F**) adult eye. *ey* > *M1BP*^*RNAi*^ exhibits a range of eye suppression phenotype ranging from a (**C**,**D**) small-eye to a "no-eye". (**G**) The area of adult eye was quantified using Image J software (NIH). The p values for the eye size (μm^2^) were calculated in a set of five (n = 5) using Student’s t-test in MS Excel Software. e*y-*Gal4 was found to be statistically significant from *ey* > *M1BP*^*RNAi*^ in case of both small-eye (p < 0.001, ***) and no-eye phenotype (p < 0.001, ***). The orientation of all imaginal discs is identical with posterior to the left and dorsal up. The magnification of all eye-antennal imaginal disc is 20 × and the adult eye is 10 ×. A total of five eye-antennal imaginal discs (n = 5) for each genotype were analyzed for respective immunohistochemistry staining.

The larval eye imaginal disc (Fig. 1A) develops into the adult compound eye comprising of 600–800 ommatidia or unit eyes (Fig. 1B). Targeted misexpression of UAS-GFP reporter transgene under *ey*-Gal4 driver (*ey* > GFP, shown in green) marks the entire eye imaginal disc (Fig. 1A). The eye discs were stained with a membrane-specific marker Dlg and pan-neuronal marker Elav (red), which marks the nuclei of the photoreceptor neurons. Targeted misexpression of inducible UAS-*M1BP*^*RNAi*^ transgene using *ey*-Gal4 driver *(ey* > *M1BP*^*RNAi*^*)*, which downregulates M1BP function in the developing eye imaginal disc, results in the suppression of eye fate (Fig. 1C,E). The eye suppression phenotype of *ey* > *M1BP*^*RNAi*^ is evident from pan-neuronal marker Elav expression, which results in either highly reduced eye field (Fig. 1C) or a “no-eye” phenotype (Fig. 1E). The adult flies of *ey* > *M1BP*^*RNAi*^ genotype also exhibits reduced eye phenotype (Fig. 1D). The penetrance of eye phenotype(s) in the adult ranges from “small-eye” (Fig. 1D, 5%, n = 100) to a “no-eye” (Fig. 1F, 95%, n = 100). Further, quantification of the adult eye area shows that the eye size significantly reduces in case of both small-eye as well as “no-eye” phenotype in *ey* > *M1BP*^*RNAi*^ flies, when compared with the control (*ey* > GFP) flies (p < 0.001, Fig. 1G). We also studied the M1BP gain-of-function phenotype using the CRISPR/Cas9- based transcriptional activation approach^69^. We did not see any eye phenotypes in terms of change in size or fate. Although we found higher levels of M1BP protein expressed in the *dpp*-Gal4 driver expression domain (Fig. S1). These results suggest that M1BP function is required for *Drosophila* eye development.

### Eye suppression phenotype due to downregulation of M1BP function has no domain constraint

**Figure 2:**
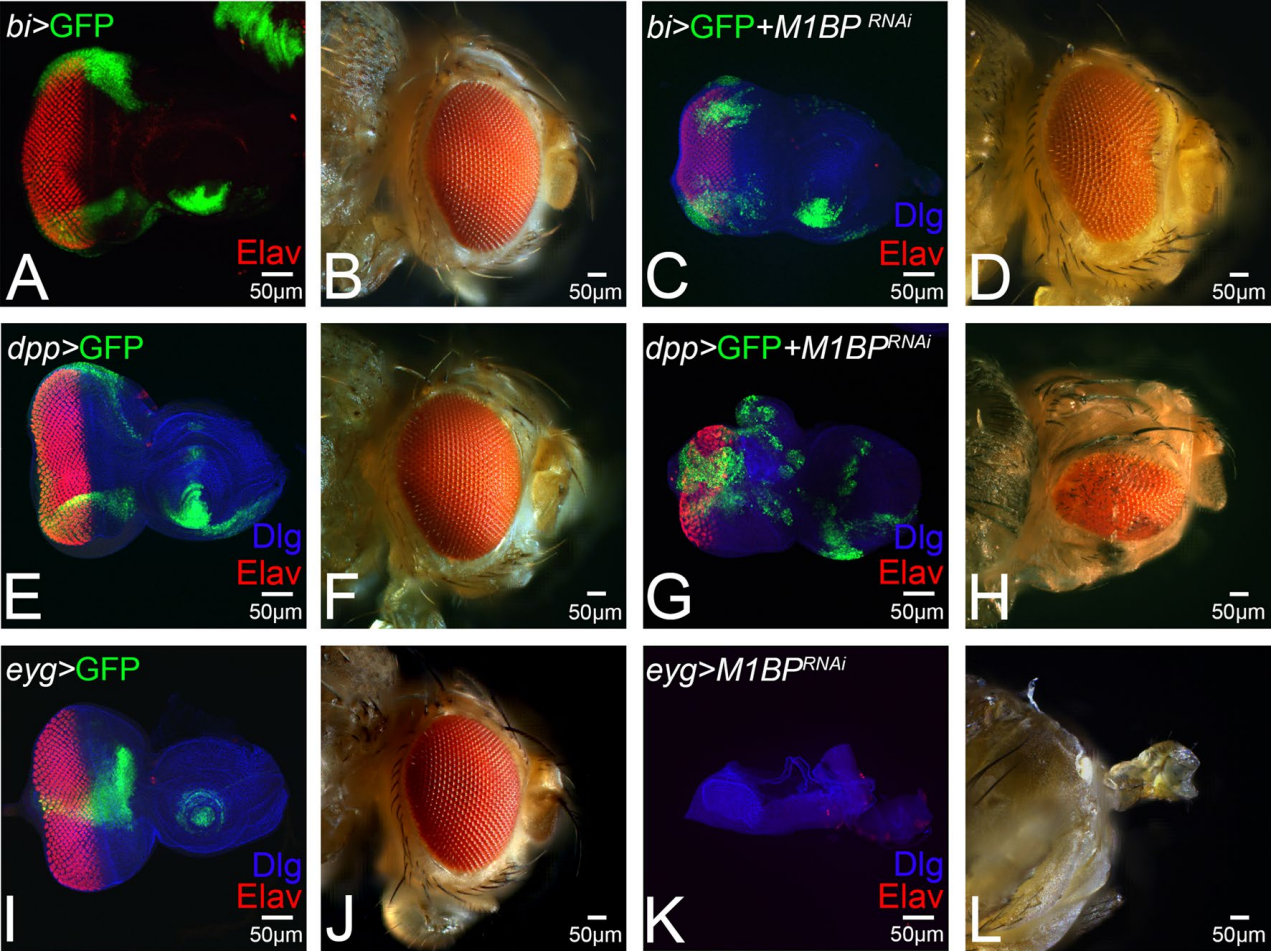
Eye suppression phenotype due to downregulation of M1BP has no domain constraint in the developing eye. Downregulation of M1BP in different domains of the developing eye using (**A**–**D**) *bi*-Gal4 driver (dorso-ventral margin, green), (**E**–**H**) *dpp*-Gal4 driver (posterior margin, green), (**I**–**L**) *eyg*-Gal4 driver (equatorial domain, green). Note that pan-neuronal marker Elav (red) marks the retinal neuron fate and GFP (green) marks the expression domain of the driver. Downregulation of M1BP using (UAS-M1BP^*RNAi*^) (**C**,**D**) in *bi-*Gal4 *(bi* > *M1BP*^*RNAi*^*)* domain results in suppression of eye fate, (**G**,**H**) in *dpp-*Gal4 *(dpp* > *M1BP*^*RNAi*^*)* domain results in eye suppression, and (**K**,**L**) in *eyg-*Gal4 domain *(eyg* > *M1BP*^*RNAi*^*)* results in complete loss of eye and head field as seen in the (**K**) eye imaginal disc and (**L**) the adult eye. The orientation of all imaginal discs is identical with posterior to the left and dorsal up. The magnification of all eye-antennal imaginal disc is 20 × and adult eye is 10 ×. A total of five eye-antennal imaginal discs (n = 5) for each genotype were analyzed for respective immunohistochemistry staining.

In order to understand if the M1BP function has any domain constraint, we downregulated M1BP functions in different domains of the developing eye by misexpressing inducible UAS-*M1BP*^*RNAi*^ transgene using various Gal4 drivers. The *bi*-Gal4 drives expression of UAS-GFP reporter (*bi* > GFP, shown in green) along the dorso-ventral (DV) margin of larval eye imaginal disc (Fig. 2A) as well as the adult eye (Fig. 2B)^64,79^. Downregulation of M1BP function in the *bi* expression domain *(bi* > *M1BP*^*RNAi*^*)* results in eye suppression along the DV margin in the eye imaginal disc (Fig. 2C). About 56% (n = 100) of the *bi* > *M1BP*^*RNAi*^ adult flies showed reduced eye phenotype (Fig. 2D). We employed *dpp*-Gal4 driver, which drives expression of UAS-GFP reporter (*dpp* > *GFP,* shown in green) along the posterior margin of the developing eye imaginal disc (Fig. 2E)^30^, and on its own it does not affect the phenotype of adult eye (Fig. 2F). Downregulation of M1BP function in the *dpp* expression domain (*dpp* > *M1BP*^*RNAi*^*)* results in reduced eye field as seen in the eye imaginal disc (Fig. 2G) and in 19% (n = 100) of the adult flies observed (Fig. 2H). Further, we used *eyg*-Gal4, which drives the expression of UAS-GFP reporter (*eyg* > GFP, shown in green) at the equator of the developing eye disc (Fig. 2I)^26^, and does not affect the eye size on its own (Fig. 2J). Downregulation of M1BP function in *eyg* expression domain results in headless phenotype (Fig. 2K,L). The frequency of headless flies were around 42% (n = 100) of the adults screened. These results clearly demonstrated that there is no domain constraint in M1BP function to promote eye development.

### Downregulation of M1BP function blocks the eye fate and MF progression

**Figure 3:**
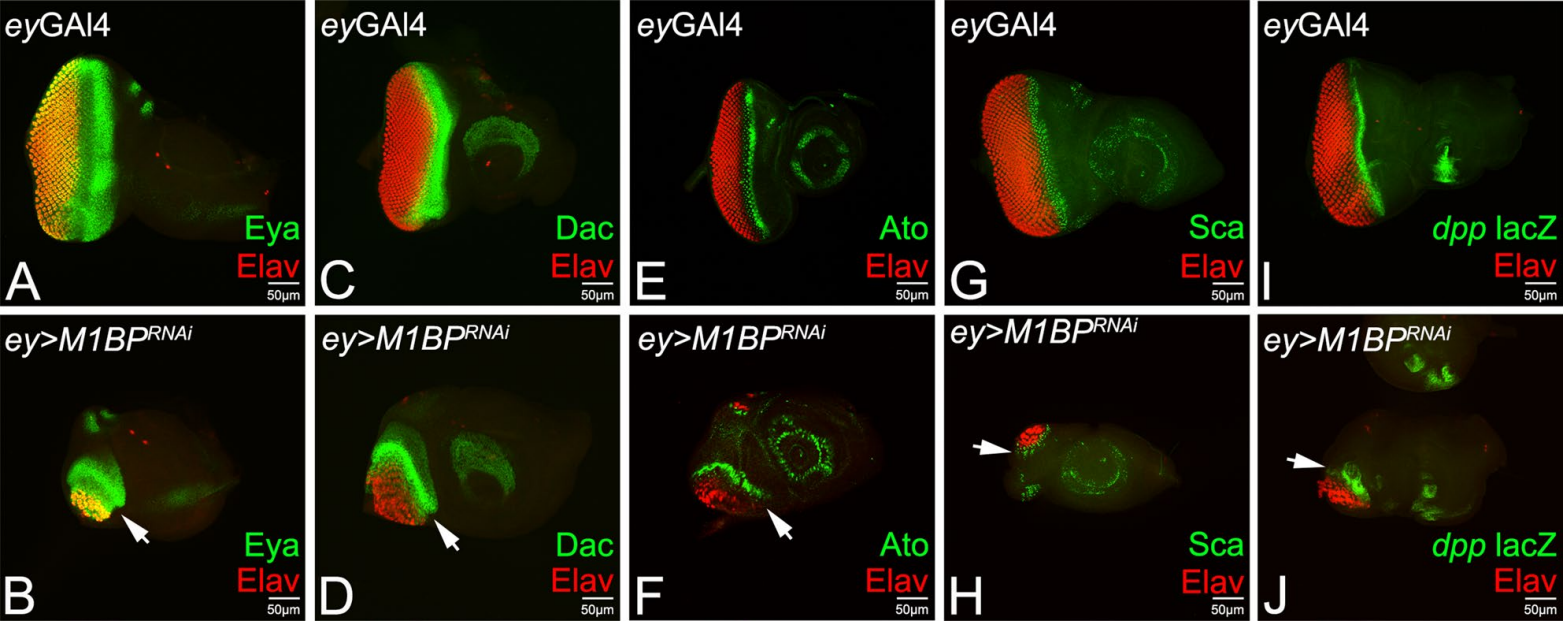
Downregulation of M1BP affects retinal determination, retinal differentiation and Morphogenetic furrow (MF) progression. Eye-antennal imaginal disc of third instar larva stained for pan-neuronal marker Elav (red), which marks the photoreceptors and retinal determination markers (**A**,**B**) Eya (green), (**C**,**D**) Dac (green), Retinal differentiation markers (**E**,**F**) Ato, (**G**,**H**) Sca, and MF marker (**I**,J) *dpp*-lacZ reporter. Note that wild-type expression of (**A**) Eya (green), (**C**) Dac (green), (**E**) Ato (green), (**G**) Sca (green) and (**I**) *dpp*-lacZ (green) is downregulated in (**B**,**D**,**F**,**H**,**J**) *ey* > *M1BP*^*RNAi*^ (arrow). Downregulation of M1BP function in the eye disc *(ey* > *M1BP *^*RNAi*^*)* suppresses expression of MF specific marker *dpp*-*lacZ* (arrow) as the eye field is reduced. The orientation of all imaginal discs is identical with posterior to the left and dorsal up. The magnification of all eye-antennal imaginal disc is 20 ×. A total of five eye-antennal imaginal discs (n = 5) for each genotype were analyzed for respective immunohistochemistry staining.

Since downregulation of M1BP causes eye suppression, we studied Retinal Determination (RD) gene expression levels as a read-out to study retinal determination and differentiation, the fundamental processes in the developing eye. A RD gene, *eya*, which acts downstream to *ey*, is expressed in a broader stripe in the differentiated cells posterior to the MF (Fig. 3A)^80^ whereas *dac* is expressed as two stripes directly anterior and posterior to the MF (Fig. 3C)^81^. We found that the downregulation of M1BP in the entire eye disc using *ey*-Gal4 *(ey* > *M1BP*^*RNAi*^*)* significantly reduces the size of the eye field as evident from the pan-neuronal marker Elav expression, which was accompanied by strong suppression of Eya and Dac expression levels (Fig. 3B,D, arrows).

Expression of Atonal (Ato) and Scabrous (Sca) serves as early markers for retinal differentiation and are employed for R8 specification (Fig. 3E,G)^32,82,83^. Based on Elav and RD gene expression, we found that misexpression of UAS-*M1BP*^*RNAi*^ in the developing eye *(ey* > *M1BP*^*RNAi*^*)* suppresses retinal neuron(s) differentiation as evident from significantly reduced expression levels of Ato and Sca (Fig. 3F,H, arrows). Our data suggests that downregulation of M1BP function not only affects the retinal determination but also suppresses the markers for R8 photoreceptor differentiation. It is known that R8 specification and differentiation is associated with MF progression. We therefore tested the requirement of M1BP function in MF progression.

In the developing eye imaginal disc, Hh and Dpp signaling is required for normal initiation and progression of MF^14,29,84,85^. We used *dpp*-lacZ, a transcriptional reporter for *dpp* gene, which also marks the progression of MF in the third instar eye imaginal disc. *dpp*-lacZ is expressed in a thin stripe that overlays the apical constrictions caused by the MF cells and marks the anterior boundary of Elav positive differentiated retinal neurons (Fig. 3I). *dpp-*lacZ expression in *ey* > *M1BP*^*RNAi*^ discs shows that MF fails to progress from the posterior margin of the eye disc towards the anterior side (Fig. 3J, arrow) and hence, downregulation of M1BP in the developing eye represses differentiation, resulting in eye suppression. To discern the mechanism behind eye suppression phenotypes of *ey* > *M1BP*^*RNAi*^*,* we looked for the putative target(s) of M1BP.

### Downregulation of M1BP function induces *wg* and Hth expression

**Figure 4:**
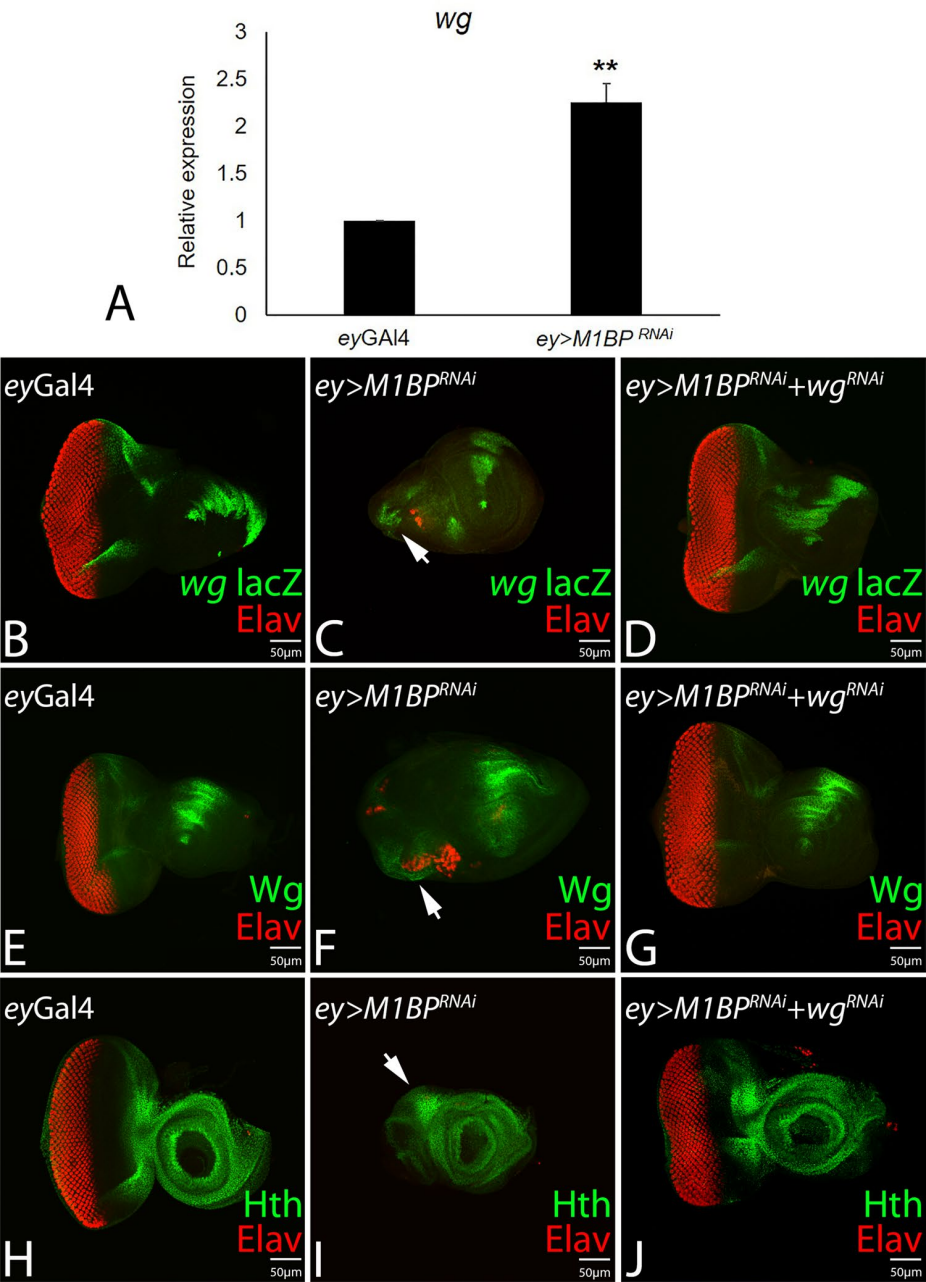
Downregulation of M1BP induces ectopic expression *wg* and *hth* expression in the developing eye disc. (**A**) Relative expression of *wg* at the transcriptional level using quantitative PCR (q-PCR) in ey-Gal4, and *ey* > *M1BP*^*RNAi*^ background. (**B**,**E**,**H**) Eye antennal imaginal disc stained for (**B**) *wg-lacZ* reporter (green) (**E**) Wg protein exhibits antero-lateral expression, (**H**) Hth (Green), a negative regulator of eye, is expressed anterior to the MF in the eye disc. (**C**,**F**,**I**) Downregulation of M1BP (*ey* > *M1BP*^*RNAi*^) suppresses the eye fate accompanied with ectopic induction of (**B**) *wg* transcription as evident from lacZ reporter (arrow), (**E**) Wg protein (arrow), and (**I**) Hth, (arrow) in the developing eye field. (**D**,**G**,**J**) Downregulation of *wg* by using *ey* > *wg*^*RNAi*^ along with *M1BP*^*RNAi*^ (*ey* > *M1BP*^*RNAi*^ + *wg*^*RNAi*^) restores reduced eye size and (**D**) *wg*-lacZ, (**G**) Wg protein, and (**J**) Hth expression in the eye imaginal disc. The orientation of all imaginal discs is identical with posterior to the left and dorsal up. The magnification of all eye-antennal imaginal disc is 20 ×. A total of five eye-antennal imaginal discs (n = 5) for each genotype were analyzed for respective immunohistochemistry staining.

In the developing eye imaginal disc, Wg serve as a negative regulator of eye fate, and blocks the progression of MF^16,37,38,85,86^. We tested if downregulation of M1BP affects the *wg* gene expression. Since M1BP is a transcriptional pausing factor, we studied the *wg* gene transcription quantitatively by using qPCR approach and qualitatively by using *wg-*lacZ reporter. The qPCR data showed that there is 2.2 fold increase in *wg* mRNA levels in *ey* > *M1BP*^*RNAi*^ discs as compared to the controls (Fig. 4A).

The *wg-lacZ* reporter is expressed at antero-lateral margins of the developing third instar larval eye imaginal disc (Fig. 4B, shown in green). The *ey* > *M1BP*^*RNAi*^ eye imaginal discs are significantly reduced in size and exhibits a robust ectopic induction of *wg*-lacZ reporter (arrow, Fig. 4C). To test if *wg* upregulation in *ey* > *M1BP*^*RNAi*^ discs is responsible for eye suppression phenotype, we downregulated *wg* gene expression levels using *wg*^*RNAi*^ in the background of *ey* > *M1BP*^*RNAi*^ (*ey* > *M1BP*^*RNAi*^ + *wg*^*RNAi*^), which resulted in significant reduction in eye suppression phenotype and restoration of size of eye field to near wild-type (Fig. 4D). In addition *wg-lacZ *reporter expression is restored (Fig. 4D). In comparison to the wild-type Wg expression in eye disc (Fig. 4E), we found robust induction and ectopic localization of Wg protein in reduced eye disc of *ey* > *M1BP*^*RNAi*^ (arrow, Fig. 4F) whereas Wg protein levels are restored to wild-type levels in *ey* > *M1BP*^*RNAi*^ + *wg*^*RNAi*^ background (Fig. 4G).

Since Wg is a negative regulator of eye development and it promotes head fate by inducing downstream *hth* expression, we further analyzed Hth protein localization in the eye discs of *ey*-Gal4 (Fig. 4H), *ey* > *M1BP*^*RNAi*^ (Fig. 4I) and *ey* > *M1BP*^*RNAi*^ + *wg*^*RNAi*^ background(s) (Fig. 4J). Hth, which is predominantly expressed anterior to the MF (Fig. 4H)^39,47,48^, exhibits robust induction in the reduced eye field of *ey* > *M1BP*^*RNAi*^ (arrow, Fig. 4I). We found that downregulation of M1BP in the eye disc induces robust Hth expression. These results strongly imply that M1BP plays an important role in promoting eye development by negatively regulating Wg and downstream Hth levels in the developing eye.

### Reducing *hth* function rescues M1BP loss-of-function phenotype in developing eye

**Figure 5:**
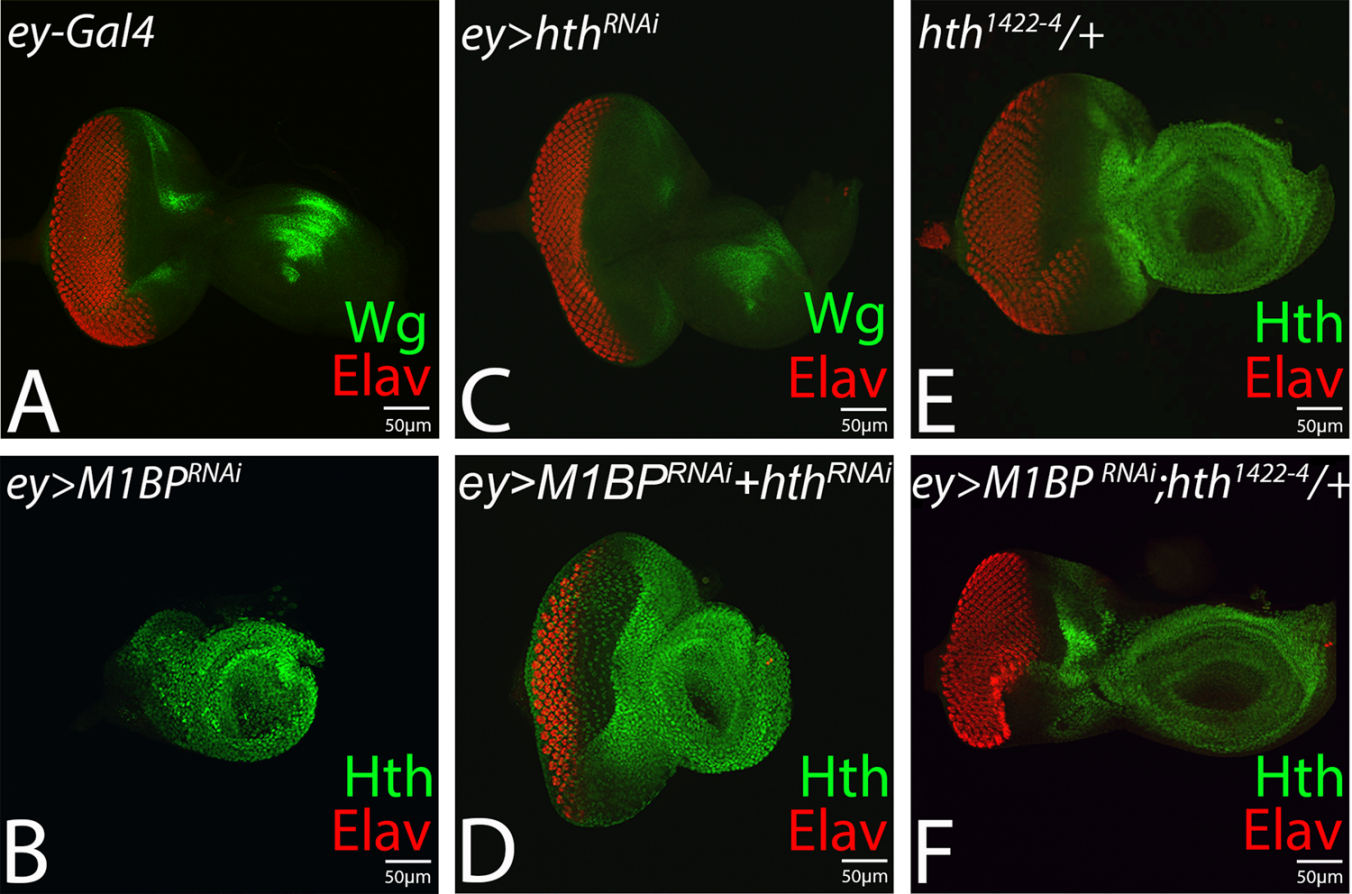
Downregulation of *hth* rescues M1BP loss-of-function phenotype of reduced eye. The reduced eye phenotype of (**B**) *ey* > *M1BP*^*RNAi*^ is rescued when *hth* levels are downregulated using (**D**) *hth*^*RNAi*^* (ey* > *M1BP*^*RNAi*^ + *hth*^*RNAi*^) or heterozygous combination of (**F**) *hth*^*1422–4*^ (*ey* > *M1BP*^*RNAi*^ + *hth*^*122–4*^* /* +*)*, a null allele of *hth*. Eye-antennal disc of (**A**) *ey-*Gal4, (**B**) *ey* > *M1BP*^*RNAi*^**,** (**C**) *ey* > *hth*^*RNAi*^**,** (**D**) *ey* > *M1BP*^*RNAi*^ + *hth*^*RNAi*^*.* (**E**) *ey* > *hth*^*RNAi*^, (**F**) *ey* > *M1BP*^*RNAi*^ + *hth*^*RNAi*^ .

We wanted to determine, if reduced eye or no-eye phenotype observed in *ey* > *M1BP*^*RNAi*^ background, is due to induction of *hth* or if there is/are other downstream target(s) of Wg signaling. Therefore, we reduced *hth* levels using inducible UAS-*hth*^*RNAi*^ or a heterozygous background of *hth* null allele*, hth*^*1422–4*^*/TM6BTb*^47^. In comparison to the control eye imaginal disc (Fig. 5A), downregulation of M1BP (*ey* > *M1BP*^*RNAi*^*)* results in a highly reduced “no-eye” phenotype (Fig. 5B), whereas downregulation of *hth* levels using UAS-*hth*^*RNAi*^ in *ey* > *M1BP*^*RNAi*^ background (*ey* > *M1BP*^*RNAi*^ + *hth*^*RNAi*^) results in a significant rescue of *ey* > *M1BP*^*RNAi*^ phenotype (Fig. 5D). In a heterozygous combination, the null allele of *hth*, *hth*^*1422–4*^*/TM6B,* exhibits a normal eye phenotype (Fig. 5E). However, reduction of *hth* function in *ey* > *M1BP*^*RNAi*^ background (*ey* > *M1BP*^*RNAi*^, *hth*^*1422–4*^*/TM6B)* exhibits significant rescue of “no-eye” phenotype (Fig. 5F)*.* However, there is no complete rescue to a wild-type eye and the frequency is low*.*

### Downregulation of M1BP triggers developmental cell death

**Figure 6:**
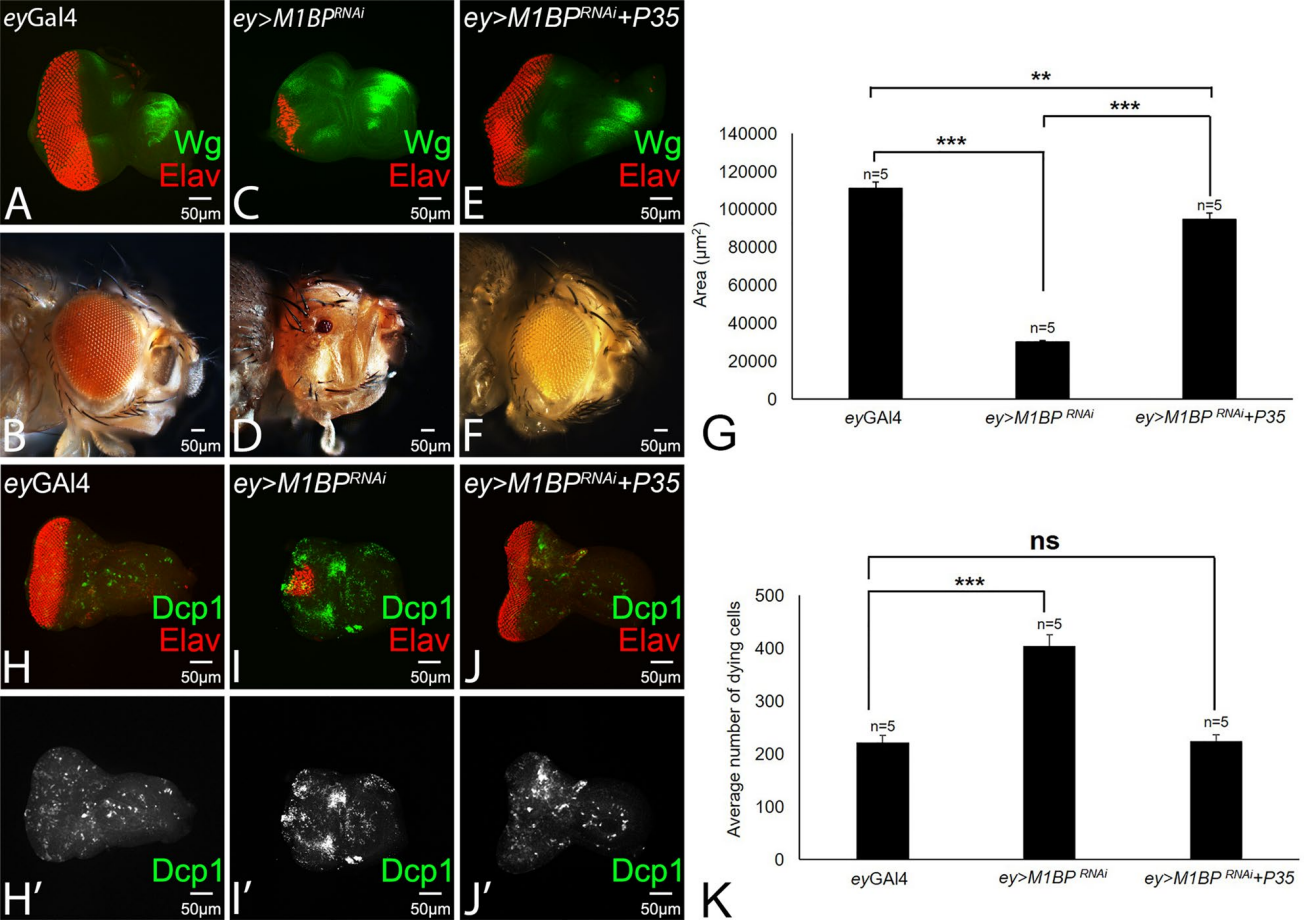
Blocking cell death can rescue eye suppression phenotype of M1BP loss-of-function. (**A**, **B**) Wild-type eye imaginal disc and the adult eye. Note that eye imaginal disc is stained for Elav (red) and Wg (green). (**C**) Loss-of-function of M1BP (*ey* > *M1BP*^*RNAi*^) results in suppression of eye fate as seen in the eye disc (**D**) adult eye. Blocking cell death by ectopic expression of P35 in *ey* > *M1BP*^*RNAi*^ background can rescue the eye suppression phenotype as seen in the eye disc and the adult eye. (**G**) The area of the adult eye was quantified using ImageJ software (NIH). The p values for the eye size (μm^2^) were calculated in a set of five (n = 5) using Student’s t-test in MS Excel Software. The eye size in *ey-*Gal4 flies were found to be highly significant from *ey* > *M1BP*^*RNAi*^ (p < 0.001, ***) than *ey* > *M1BP*^*RNAi*^ + *P35* flies (p < 0.01, **). Blocking cell death in *ey* > *M1BP*^*RNAi*^ + *P35* flies restores the eye size, when compared with the *ey-*Gal4 flies (p < 0.05, *). (**H**–**J**) Eye imaginal disc stained for Elav (red) and Dcp-1 (green). Note that Dcp-1 marks the dying cells in the disc. (**H’**–**J’**) Eye antennal imaginal disc showing split channel for Dcp-1 staining. (**K**) Quantification of the Dcp-1 positive cells (green) shows that the average number of dying cells were significantly higher in *ey* > *M1BP*^*RNAi*^ (p < 0.001, ***) than *ey-*Gal4 discs, however, when compared with the *ey* > *M1BP*^*RNAi*^ + *P35* discs, the number of dying cells were found to be comparable (non-significant) with the *ey-*Gal4 discs. The orientation of all imaginal discs is identical with posterior to the left and dorsal up. The magnification of all eye-antennal imaginal disc is 20 × and the adult eye is 10 ×. A total of five eye-antennal imaginal discs (n = 5) for each genotype were analyzed for respective immunohistochemistry staining.

It has been shown that ectopic induction of Wg signaling in the eye disc induces developmental cell death, which results in reduced eye phenotypes^54^. To understand the genetic mechanism responsible for the reduced eye phenotype, manifested by flies where M1BP function was downregulated, we tested the role of cell death. It is known that ectopic expression of baculovirus P35 blocks caspase-dependent cell death^56^. In comparison to the wild-type eye-antennal imaginal disc and the adult eye (Fig. 6A,B), downregulation of M1BP function in *ey* > *M1BP*^*RNAi*^ results in reduced eye as seen in the eye imaginal disc and the adult eye (Fig. 6C,D). Blocking caspase-dependent cell death by ectopic expression of UAS-*P35* transgene in *ey* > *M1BP*^*RNAi*^ (*ey* > *M1BP*^*RNAi*^ + *P35*) background can restore the eye suppression phenotype as observed in the eye disc and the adult eye (Fig. 6E,F). Further, quantification of the area of the adult eyes of *ey*-Gal4 (Fig. 6B,G), *ey* > *M1BP*^*RNAi*^ (Fig. 6D,G) and *ey* > *M1BP*^*RNAi*^ + *wg*^*RNAi*^ background(s) (Fig. 6F,G) shows that the eye size significantly reduces in *ey* > *M1BP*^*RNAi*^ flies, when compared with *ey*-Gal4 (p < 0.001, Fig. 6G). However, blocking caspase-dependent cell death significantly restores the eye size in *ey* > *M1BP*^*RNAi*^ + *P35* background, when compared with the *ey* > *M1BP*^*RNAi*^ flies (p < 0.001, Fig. 6G), and less significant than control *ey-*Gal4 flies (p < 0.01, Fig. 6G).

To validate our hypothesis that ectopic upregulation of *wg* induces developmental cell death, which results in reduced eye phenotype seen in *ey* > *M1BP*^*RNAi*^ eye disc, we used the antibody against Drosophila effector caspase, death caspase-1 (Dcp-1). Dcp-1, a critical executioner of apoptosis, serves as an excellent marker for cell death^87^. In the control *ey*-Gal4 eye disc, we found a few Dcp-1 positive dying cells/ retinal neurons (Fig. 6H,H’). The number of Dcp-1 positive dying cells gets almost doubled in *ey* > *M1BP*^*RNAi*^ eye disc, which is highly reduced in size (Fig. 6I,I’,K). The number of Dcp-1positive dying cells is restored to the control ey-Gal4 (Fig. 6K) when *P35* levels are upregulated in *ey* > *M1BP*^*RNAi*^ (*ey* > *M1BP*^*RNAi*^ + *P35)* background (Fig. 6J,J’). Further, quantification of the Dcp-1 positive nuclei shows that downregulation of M1BP in the eye disc (*ey* > *M1BP*^*RNAi*^) induces apoptotic cell death as the average number of dying cells were significantly higher in *ey* > *M1BP*^*RNAi*^ (p < 0.001, Fig. 6K) as compared to the control *ey-*Gal4 eye discs, however, when compared with the *ey* > *M1BP*^*RNAi*^ + *P35* discs, the number of Dcp-1 positive dying cells were non-significant with respect to the *ey-*Gal4 eye discs (ns, Fig. 6K). These results suggest that overexpressing P35 where M1BP levels are downregulated restores the size of the eye field by reducing the average number of dying cells.

### Downregulation of M1BP is independent of cell proliferation function

**Figure 7:**
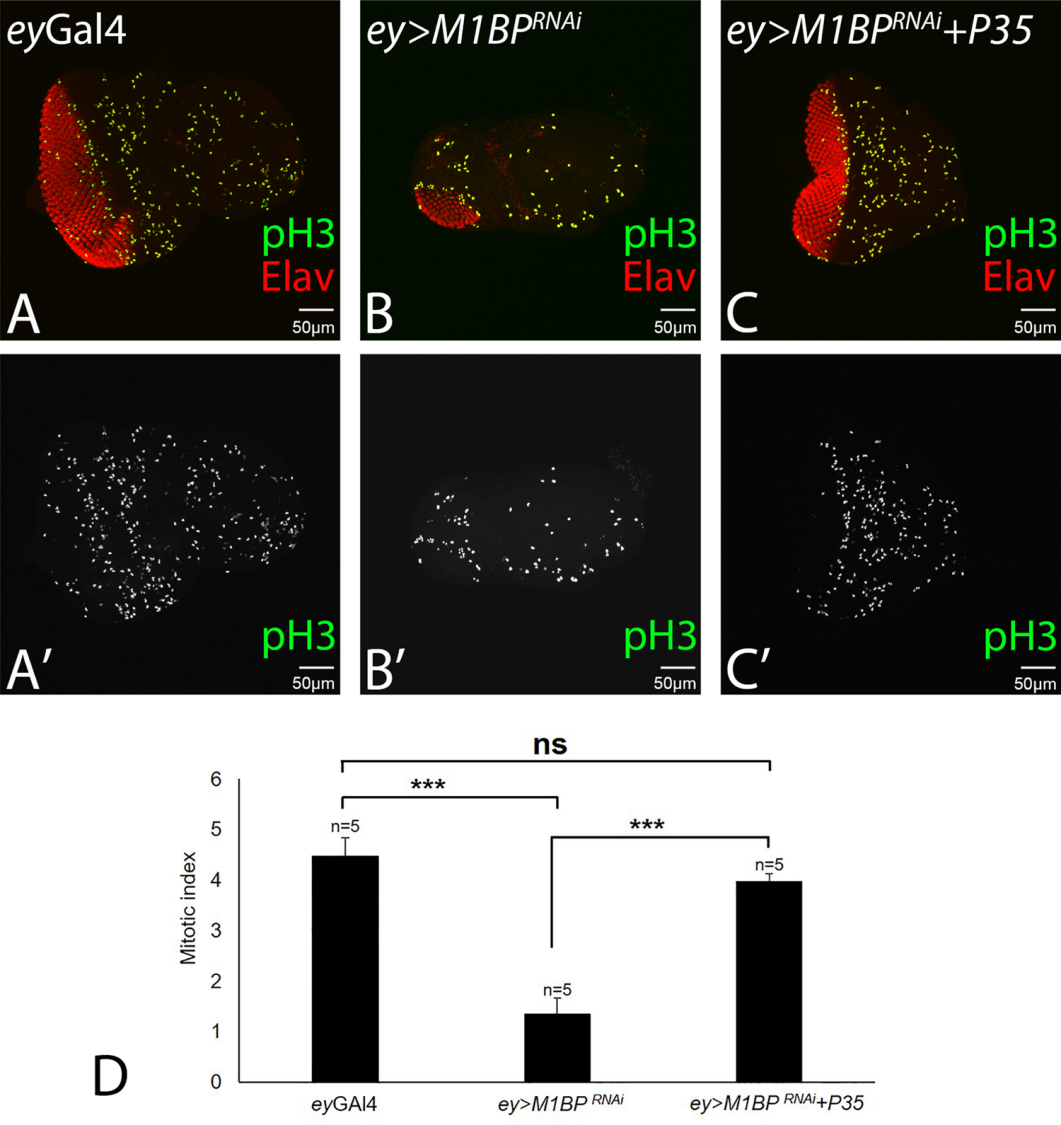
Overexpression of P35 in loss-of-function background of M1BP promotes proliferation in the developing eye field. (**A**–**C**) Eye imaginal disc stained for Elav (red) and pH3 (green). Note that pH3 marks the proliferating cells in the disc. (**A’**–**C’**) Eye antennal imaginal disc showing split channel for pH3 staining. (**D**) Quantification of the pH3 positive cells shows (green) that the proliferating cells are significantly reduced in *ey* > *M1BP*^*RNAi*^ (p < 0.001, ***) than *ey*Gal4 discs, however, when compared with the *ey* > *M1BP*^*RNAi*^ + *P35* discs, the number of proliferating cells were found to be comparable (non-significant) with the *ey-*Gal4 discs. Overexpression of P35 in M1BP loss-of-function background promotes significantly higher rate of proliferation (p < 0.001, ***), when compared with the *ey* > *M1BP*^*RNAi*^ discs. The orientation of all imaginal discs is identical with posterior to the left and dorsal up. The magnification of all eye-antennal imaginal disc is 20 ×. A total of five eye-antennal imaginal discs (n = 5) for each genotype were analyzed for respective immunohistochemistry staining.

Since downregulation of M1BP function results in the small-eye phenotype, it is possible that the reduced number of Elav positive cells (red; Fig. 7B), which marks the photoreceptor neurons in the eye disc, is due to reduced cell proliferation. To test the role of cell proliferation in reduced eye phenotype, we stained the eye imaginal discs with phospho-histone 3 (pH3) that marks the proliferating cells (Fig. 7). Quantification of the pH3 positive cells show that the proliferating cells are significantly reduced in *ey* > *M1BP*^*RNAi*^ discs (Fig. 7B,B’,D; p < 0.001) when compared with *ey-*Gal4 discs (Fig. 7A,A’,D). The reduction in number of pH3 positive cells in *ey* > *M1BP*^*RNAi*^ discs does not clearly address if both cell death and cell-proliferation are involved. Although we have seen earlier that reduced size of the *ey* > *M1BP*^*RNAi*^ eye disc is due to developmental cell death. In order to test the role of cell proliferation, we counted the pH3 positive cells in the *ey* > *M1BP*^*RNAi*^ + *P35* eye disc (where caspase-dependent cell death is blocked). Overexpression of UAS-P35 transgene along with downregulation of M1BP (*ey* > *M1BP*^*RNAi*^ + *P35*) results in significant increase in the number of proliferating cells *ey* > *M1BP*^*RNAi*^ + *P35* discs, when compared with *ey* > *M1BP*^*RNAi*^ discs (p < 0.001, Fig. 7C,C’, D). Interestingly, the number of pH3 positive nuclei are restored to the control (Fig. 7A,D). This data suggests that cell proliferation function is not the major contributing factor in reduced eye phenotype in *ey* > *M1BP*^*RNAi*^.

## Discussion

Pol II pausing near the transcription start site has been identified as a key step in optimizing transcription of many genes in metazoans. It has been proposed that pausing allows the coupling of transcription and RNA processing^88^. Pausing can contribute to dynamic regulation of gene expression in response to developmental and environmental signals^7,89^, and can function to repress transcription^90^. The genome-wide studies have revealed that ~ 10–40% of all genes in mammalian embryonic stem cells and *Drosophila* have paused promoters^2,91–93^. In *Drosophila*, while the phenomenon of promoter proximal pausing has been well studied in regulation of genes encoding the heat shock proteins (Hsp) and different components involved in immune response pathways^6,90,94^, it is also proposed to play important role in regulating the gene expression during early developmental events such as patterning, sex determination etc.^2,5,7,95^. So far, the sequence-specific transcription factors such as GAGA factor and M1BP, and other regulators HEXIM, LARP7 (La Ribonucleoprotein 7, Transcriptional Regulator) have been implicated in dictating Pol II pausing in *Drosophila*^57,96^. However, the biological relevance of transcriptional pausing and the exact mechanism by which the regulatory factors may contribute in pausing of Pol II is not fully understood.

### M1BP regulates retinal determination and MF progression in developing eye

We tested for the first time the role of transcription pausing factor, M1BP during *Drosophila* eye development. We found that downregulation of M1BP levels in the developing eye results in strong suppression of eye fate (Fig. 1C–F), however, gain-of-function of M1BP did not affect the eye fate (Fig. S1) suggesting that optimum levels of M1BP are required for *Drosophila* eye development. Furthermore, we did not find any domain constraint in eye suppression function when M1BP levels were downregulated (Fig. 2C,D,G,H,K,L). In addition, when M1BP levels were downregulated (*ey* > *M1BP*^*RNAi*^) the expression of retinal determination and differentiation genes were strongly downregulated (Fig. 3B,D,F,H). Interestingly, we found that protein encoded by RD genes were downregulated in *ey* > *M1BP*^*RNAi*^ background. Therefore, M1BP may not be affecting RD gene expression directly.

During eye development, a wave of differentiation, emanates from the posterior margin of the developing eye imaginal disc, which sweeps anteriorly across the retinal primordium. The crest of this wave is referred to as the MF, which results in retinal differentiation behind it^14,85^. The two signals *dpp* and *hh* plays an important role in initiation and progression of MF. We found that downregulation of M1BP affects retinal differentiation as well as progression of MF (Fig. 3). It suggests that M1BP role is to promote retinal differentiation as well as MF progression. Also, M1BP downregulates the level of negative regulator(s) of the eye fate. We screened for the genes, which may serve as target for M1BP mediated transcriptional pausing mechanism in *Drosophila* eye imaginal disc.

### M1BP regulates *wg* gene expression in the developing eye

**Figure 8:**
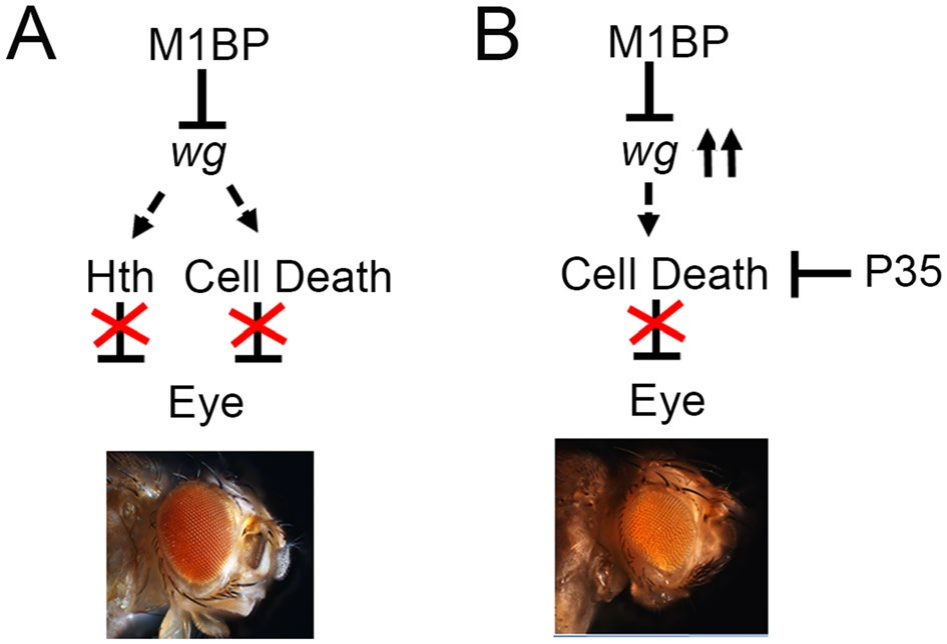
Models for M1BP function during *Drosophila* eye development. (**A**) M1BP suppresses the head fate by downregulating Wg and downstream Hth in the developing eye. Note that Wg and Hth are negative regulators of the eye development. (**B**) Blocking caspase-dependent cell death by overexpressing anti-apoptotic *P35* transgene in *ey* > *M1BP*^*RNAi*^ (*ey* > *M1BP*^*RNAi*^ + *P35*) background significantly rescues the reduced eye phenotype.

The protein encoded by *Drosophila wg* gene, a member of Wg/WNT signaling pathway, act short range inducer, which organizes the pattern of cells at a distance in the embryo. Since M1BP downregulation resulted in blocking retinal differentiation and MF progression, we looked for the targets of M1BP transcriptional pausing function using the candidate gene approach. We found that *wg*-lacZ reporter, which serves as a transcriptional read out for Wg, exhibits robust induction in eye imaginal discs where M1BP levels were downregulated (Fig. 4C). This observation was further validated by qPCR approach which showed that there is a 2.2-fold increase in *wg* gene expression (Figs. 4A, 8A). Furthermore, in high throughput microarray screen carried out in S2R + cells, *wg* was also identified as a target whose expression is downregulated by M1BP using M1BP RNAi. According to microarray analysis, wg shows a 5.5-fold change (raw value against *wg *gene ID) when cells are treated with *M1BP*^*RNAi*^ ^57^.

To validate the results from qPCR approach as well induction of *wg-*lacZ reporter expression in *ey* > *M1BP*^*RNAi*^ eye imaginal disc (Fig. 4), we also employed bioinformatics analysis to determine if there are M1BP binding sites in the *wingless (wg)* gene. The M1BP binding sequence (YGGTCACACTR) has been reported earlier^57,61^. We used this sequence for MEME analysis to screen for M1BP binding sites in *wg* gene and regulatory region^60^. We found 36 potential binding sites for M1BP in *wingless* gene and regulatory regions as shown in (S. Fig. 2B, Supplementary Table S1). Using these all 36 potential binding sites web logo was generated from weblogo.berkeley.edu/logo.cgi (Fig. S2B).

Wg, a ligand for evolutionarily conserved Wg/WNT signaling pathway, is known to act as a negative regulator of eye development^13,37–39^. During *Drosophila* eye development, Wg activity promotes head specific fate by negatively regulating MF progression in the differentiating eye imaginal disc^22,37,38^. Wg regulates expression of downstream gene *hth*, which encodes a MEIS class of transcription factor, and act as a negative regulator of eye development (Figs. 4, 8A)^12,47–49^. We found that in *ey* > *M1BP*^*RNAi*^ background, robust induction of *wg* transcription also accompanies ectopic induction of *hth* along with the suppression of the eye fate (Figs. 4I, 8). Further, downregulation of *wg* levels, using *wg*^*RNAi*^, in *ey* > *M1BP*^*RNAi*^ background rescued the eye suppression phenotype (Figs. 4, 8). This data clearly suggested that M1BP downregulates levels of *wg*, which in turn regulate expression of *hth* in the developing *Drosophila* eye (Fig. 5).

### M1BP blocks Wg upregulation mediated developmental cell death

Higher levels of Wg are known to trigger developmental cell death in the developing eye field^54^. Interestingly, in *ey* > *M1BP*^*RNAi*^ eye discs, the eye field was significantly reduced. Since, majority of the cell death is triggered by the activation of caspase-dependent cell death, blocking caspase-dependent cell death by ectopic expression of anti-apoptotic *P35* transgene^56^ in *ey* > *M1BP*^*RNAi*^ background showed rescue of eye suppression phenotype (Fig. 6E,F). However, these P35 mediated rescues of *ey* > *M1BP*^*RNAi*^ were not as significant as seen with *wg*^*RNAi*^ (Fig. 3). This suggests that Wg might be regulating eye fate through *hth* induction (Figs. 4I, 8A) and eye field size by triggering caspase mediated cell death (Figs. 6, 8B). In order to rule out that these in *ey* > *M1BP*^*RNAi*^ phenotypes are not affected by reduced cell proliferation rates, we also tested levels of pH3 in these developing eye fields (Fig. 7). We found that cell proliferation rates were not affected by this transcriptional pausing mechanism in the developing eye.

Our results imply that the transcription pausing function of M1BP in regulating Wg signaling may play a critical role in *Drosophila* eye development (Fig. 8). However, other factors and signaling pathways involved in regulating the M1BP function at the mechanistic level is yet to be determined. In order to further understand, if M1BP mediated transcriptional regulation is also implicated during development of other imaginal discs in *Drosophila*, we studied the downregulation of M1BP function in *bi-*Gal4 domains of wing imaginal disc (Fig. S3). We wanted to test if this role of M1BP in regulating *wg* gene expression is exclusive to developing eye disc or it extends to other larval imaginal disc. We employed a *bi*-GA4 driver which drives the expression of a transgene in wing imaginal disc (Fig. S2A, A” shown in green)^63,64^. Downregulation of M1BP in *bi-*Gal4 expression domains of wing (*bi* > *M1BP*^*RNAi*^, Fig. S2B, B”) exhibits ectopic upregulation *wg* expression in the pouch region of the wing imaginal disc (Fig. S2B’, arrowhead). Furthermore, M1BP expression levels are downregulated in the wing pouch region, which corresponds to the *bi-*Gal4 expression domain. These results suggested that the transcription pausing function of M1BP may have similar target in the eye and wing imaginal disc. Recently, HEXIM, another transcriptional regulator associated with pol II pausing, has been reported to affect wing development in *Drosophila* by regulating Hh signaling^97^. In *Drosophila* wing imaginal disc, HEXIM knockdown causes developmental defects by inducing ectopic expression of *hh* and its transcriptional effector *cubitus interuptus (ci)*, which triggers apoptosis. This suggests that the regulatory factors involved in Pol II pausing are important in maintaining the expression levels of different signaling pathways during development in *Drosophila*.

A number of highly conserved transcriptional pausing and elongation factors such as Spt5 precisely regulate transcription during *Drosophila* embryogenesis. The Spt5^W049^ missense mutation causes defects in the anterior–posterior patterning and segmental patterning during embryogenesis^98^. Interestingly, the mutant allele of Spt5 (foggy^m806^) in Zebrafish also causes multiple developmental defects such as discrete problems with pigmentation, tail outgrowth, ear formation and cardiac differentiation. These studies suggest that the regulatory mechanism in Pol II pausing during fly development are also conserved in higher organisms. The *Drosophila* compound eye shares similarities with the vertebrate eye at the level of genetic machinery as well as the processes of differentiation^99,100^. Therefore, the information generated in *Drosophila* can be extrapolated to higher organisms^11,100,101^. Since Wnt signaling is known to induce programmed cell death in patterning the vasculature of the vertebrate eye^102^, it will be important to study what molecules other than M1BP can prevent Wg signaling from inducing cell death during early eye development.

## Supplementary information

Supplementary file 1
