# Supplementary material for: Motif 1 Binding Protein suppresses wingless to promote eye fate in Drosophila
Source: Sci Rep. 2020 Oct 14;10:17221. doi: 10.1038/s41598-020-73891-7 (PMC7560846; doi:10.1038/s41598-020-73891-7)
Supplement: Supplementary file 1 [file 41598_2020_73891_MOESM1_ESM.docx]

**Motif 1 binding protein suppresses *wingless* to promote eye fate in *Drosophila.***

**Akanksha Raj^1^, Anuradha Venkatakrishnan Chimata^1^, Amit Singh^12345*^**

1. Department of Biology, University of Dayton, Dayton, OH, USA
2. Premedical Program, University of Dayton, Dayton, OH, USA
3. Center for Tissue Regeneration & Engineering (TREND), University of Dayton, Dayton, OH, USA
4. Integrative Science and Engineering (ISE), University of Dayton, Dayton, OH, USA
5. Center for Genomic Advocacy (TCGA), Indiana State University, Terre Haute, IN, USA

**Running Title:** M1BP suppresses *wg* gene expression to promote eye fate

**Keywords:**

*Drosophila melanogaster*, Transcription pausing, Pol II pausing, Motif 1 Binding Protein, Wingless, Eye development, Homothorax, Retinal determination,

***Corresponding author**

Amit Singh, PhD

Department of Biology,

University of Dayton,

Dayton, OH 45469, USA.

Phone: +1-9372292894

Email: [asingh1@udayton.edu](mailto:asingh1@udayton.edu)

**Supplementary Fig. 1. Overexpression of M1BP in the *dpp* domain does not affect eye fate.** (A-B) Eye-antennal imaginal disc stained for Elav (Red) and M1BP (Green). Note that the M1BP is expressed ubiquitously in the eye-antennal disc. Eye-antennal imaginal disc showing split channel for (A’-B’) Elav and (A”-B”) M1BP. CRISPR/Cas9- based transcriptional activation approach ^1^ was used to overexpress TRiP-CRISPR Overexpression (TRiP-OE) M1BP in the *dpp* domain of the developing eye by crossing the *TOE M1BP* flies with *dpp-*Gal4*; dcas9-VPR* flies, in which the tissue-specific Gal4 directs expression of a catalytically inactive dead Cas9 (dCas9) fused to a tripartite transcriptional activator domain , VP64-p65-Rta (VPR). Similarly, this approach was used to generate M1BP Knockout (KO) in the *dpp* domain of the eye disc by crossing *TKO M1BP* flies with *dpp*Gal4*; dcas9-VPR* flies. (A) Overexpression of M1BP in *dpp* domain does not affect the eye development as indicated by the Elav expression, which marks the photoreceptor neurons. The magnification of all eye-antennal imaginal disc is 20X.

**
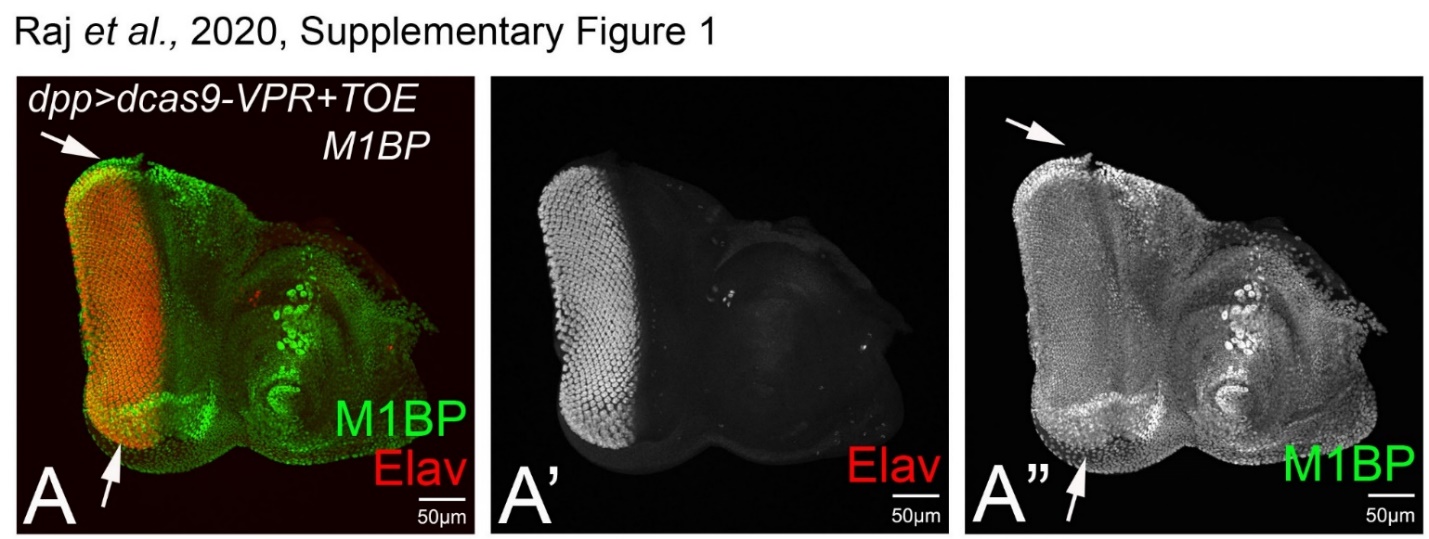
**

**Supplementary Fig. 2. Bioinformatics analysis to identify the M1BP binding sites in *wingless* gene.** (A) Motif1 consensus sequence previously identified for preferential distribution near the transcriptional start site TSS ^2^. (B) Consensus sequences identified using MEME analysis of *wingless* is similar to the previously identified sequence indicating binding capability of M1BP.

**Material and Methods for Supplementary Figure 2**

**MEME analysis protocol**

Reference sequences of *wingless* and *homothorax* were downloaded in FASTA format from NCBI database. MEME analysis was performed with these sequences and the Motif 1 consensus sequence ^2^ as inputs using the MEME-Suite ^3,4^ and the consensus sequence was generated using the WebLogo website (http://weblogo.berkeley.edu/logo.cgi).

**M1BP binding sites using JASPER**

To analyze the existing binding sites of M1BP in *Drosophila* genome, we downloaded the binding site information from JASPER 2020 ^5,6^ and visualized it using the Galaxy web tool ^7^. The UCSC table browser was used to fetch the gene list of dm6 genome assembly and the binding sites were screened.

**Supplementary Fig. 3. Downregulation of M1BP affects Wg expression in the wing imaginal disc.** *bi-*Gal4 driven expression of UAS-GFP transgene (green) in the (A, B) wing imaginal disc. The imaginal discs are stained for (A’, B’) Wg (red), (A’”, B”’,) M1BP (blue). *bi-*Gal4 expression domain is shown in green. (A”, B”) (B) Downregulation of M1BP in *bi>M1BP^RNAi^* wing imaginal disc ectopically upregulates (B’) Wg in the pouch region (Arrow) and reduced the *bi-*Gal4 driven GFP expression in *bi*-domain (Arrow). The magnification of all eye-antennal imaginal disc is 20X.

**References**

1 Ewen-Campen, B. *et al.* Optimized strategy for in vivo Cas9-activation in Drosophila. *Proc Natl Acad Sci U S A* **114**, 9409-9414, doi:10.1073/pnas.1707635114 (2017).

2 Ohler, U., Liao, G. C., Niemann, H. & Rubin, G. M. Computational analysis of core promoters in the Drosophila genome. *Genome Biol* **3**, RESEARCH0087, doi:10.1186/gb-2002-3-12-research0087 (2002).

3 Bailey, T. L. *et al.* MEME SUITE: tools for motif discovery and searching. *Nucleic Acids Res* **37**, W202-208, doi:10.1093/nar/gkp335 (2009).

4 Bailey, T. L. & Gribskov, M. Combining evidence using p-values: application to sequence homology searches. *Bioinformatics* **14**, 48-54, doi:10.1093/bioinformatics/14.1.48 (1998).

5 Fornes, O. *et al.* JASPAR 2020: update of the open-access database of transcription factor binding profiles. *Nucleic Acids Res* **48**, D87-D92, doi:10.1093/nar/gkz1001 (2020).

6 Yevshin, I., Sharipov, R., Kolmykov, S., Kondrakhin, Y. & Kolpakov, F. GTRD: a database on gene transcription regulation-2019 update. *Nucleic Acids Res* **47**, D100-D105, doi:10.1093/nar/gky1128 (2019).

7 Afgan, E. *et al.* The Galaxy platform for accessible, reproducible and collaborative biomedical analyses: 2016 update. *Nucleic Acids Res* **44**, W3-W10, doi:10.1093/nar/gkw343 (2016).

**SupplementaryTable 1: Potential MIBP binding sites (*) in regulatory region of *wingless (wg)* gene.**

| **S. No.** | **Potential M1BP binding sites in *wingless* gene** | **Locations on Chromosome 2L** |
| --- | --- | --- |
| 1 | CTGTCACAGGA | 7298249-7298259 |
| 2 | TGGCCACAAAA | 7298375-7298385 |
| 3 | TGGTCATGATG | 7298527-7298537 |
| 4 | GGGCCACACCT | 7299001-7299011 |
| 5 | TGCTCTCACAG | 7299436-7299446 |
| 6 | CTCTCACACAG | 7299589-7299599 |
| 7 | TGGCAACAATA | 7299757-7299767 |
| 8 | GGGAGACACTT | 7300334-7300344 |
| 9 | GCGTCAGACTG | 7300605-7300615 |
| 10 | TGGTGACGCCG | 7300853-7300863 |
| 11 | TAGTCACAGCG | 7301703-7301713 |
| 12 | GGATCGCACTT | 7302608-7302618 |
| 13 | CGGTAACAAAA | 7303016-7303026 |
| 14 | CGATCACAAAA | 7303933-7303943 |
| 15 | CGCCCAGACTG | 7305907-7305917 |
| 16 | TGCACAAACTG | 7306229-7306239 |
| 17 | TGGTAACAATC | 7306316-7306326 |
| 18 | CGATTACACCG | 7306805-7306815 |
| 19 | CGTTGGCACTG | 7307168-7307178 |
| 20 | ATGTTACACTT | 7308683-7308693 |
| 21 | AGGTCACAGAG | 7308952-7308962 |
| 22 | TGGAACCACTG | 7310141-7310151 |
| 23 | TGTTCATAGTG | 7310433-7310443 |
| 24 | CGATTACGCTG | 7310476-7310486 |
| 25 | TGGTGACACCT | 7310899-7310909 |
| 26 | TGCTGACGCTG | 7311111-7311121 |
| 27 | TGGCCACACCC | 7311347-7311357 |
| 28 | TGGTCTCGCTC | 7312306-7312316 |
| 29 | TGGTCGCGGTA | 7312648-7312658 |
| 30 | GGGTCGCAATC | 7313085-7313095 |
| 31 | AAGTCATACTA | 7314271-7314281 |
| 32 | CGGACATCCTG | 7314616-7314626 |
| 33 | TGGTCAAAGGG | 7314698-7314708 |
| 34 | AGCTCTCACTT | 7315364-7315374 |
| 35 | TGGTCATACAA | 7315847-7315857 |
| 36 | TAGTTAAACTA | 7316070-7316080 |

* MIBP binding site: **YGGTCACACTR**
